# Why CO_2_ capture *via* supercapacitive swing adsorption needs a theory, not just tweaks

**DOI:** 10.1039/d6sc02838a

**Published:** 2026-06-30

**Authors:** Malina Seyffertitz, Zeke Coady, Cerys Walsh, Selina E. Wiesner, Jack S. Taylor, Grace E. Mapstone, Alexander C. Forse

**Affiliations:** a Yusuf Hamied Department of Chemistry, University of Cambridge Lensfield Road, CB2 1EW UK ms3216@cam.ac.uk acf50@cam.ac.uk

## Abstract

Supercapacitive Swing Adsorption (SSA) is an emerging electrochemical approach to CO_2_ capture that uses the charging and discharging of aqueous supercapacitors with a gas-exposed electrode. Its appeal lies in low-energy operation, long cycle lifetime, humidity tolerance and the use of sustainable and abundant materials. Yet, despite a rapidly growing literature and extensive efforts to optimise the process, CO_2_ adsorption capacities remain modest compared with more established capture technologies, and the underlying capture mechanism remains unresolved. Here, we argue that further optimisation is increasingly limited by this lack of mechanistic understanding. We first map the empirical landscape of SSA, summarising robust trends across electrolytes, electrode materials, charging protocols, and gas composition. From these trends, we derive mechanistic constraints that any theory of SSA must be able to explain, and assess their compatibility with the leading mechanistic proposals (gas–solid, molecular liquid–solid, ionic liquid–solid, and pH-swing-based mechanisms), highlighting tensions and open questions. Finally, we outline possible strategies for closing these gaps and resolving the operative capture mechanism and driving forces, with the aim of enabling a transition from empirical optimisation toward predictive design of SSA systems as a viable carbon capture technology.

## Abstract 2

Supercapacitive Swing Adsorption (SSA) is an emerging electrochemical approach to CO_2_ capture that uses the charging and discharging of aqueous supercapacitors. Its further development is hindered by limited mechanistic understanding.

## Graphical abstract

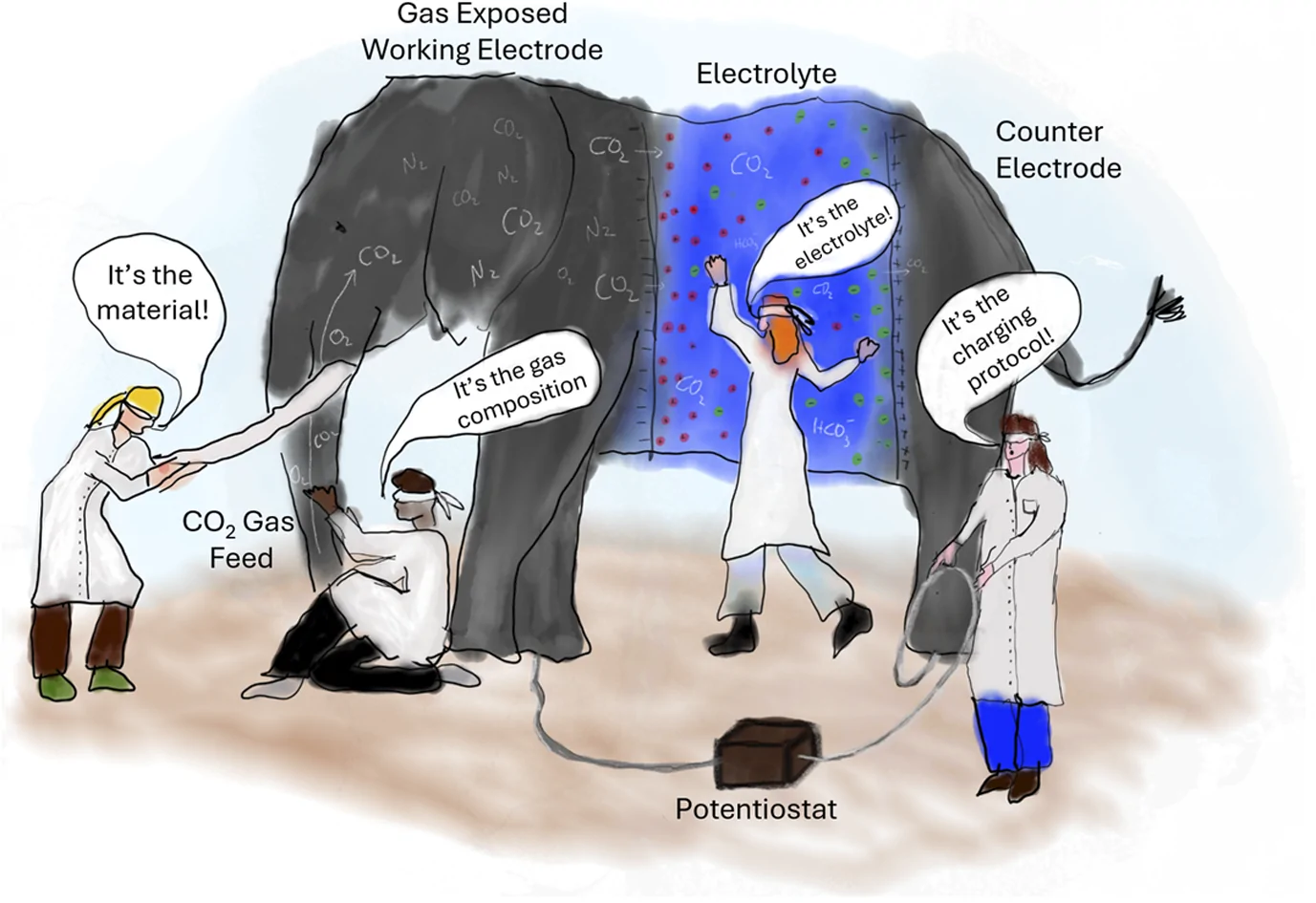

## Introduction

As of 2025, the global carbon budget for limiting warming to 1.5 °C above pre-industrial levels stands at just 130 gigatonnes of carbon dioxide,^1^ a figure that continues to shrink against the backdrop of annual emissions exceeding 40 gigatonnes.^2^ While rapid emissions reduction remains the most effective measure, most mitigation pathways now assume large-scale carbon dioxide capture, between 7 and 9 gigatonnes annually by mid-century,^3^ to compensate for hard-to-abate sectors.^3,4^ Meeting that demand will require carbon capture technologies for point-source capture and direct air capture that are not just effective, but low-cost, low-energy, and rapidly scalable.

**Fig. 1:**
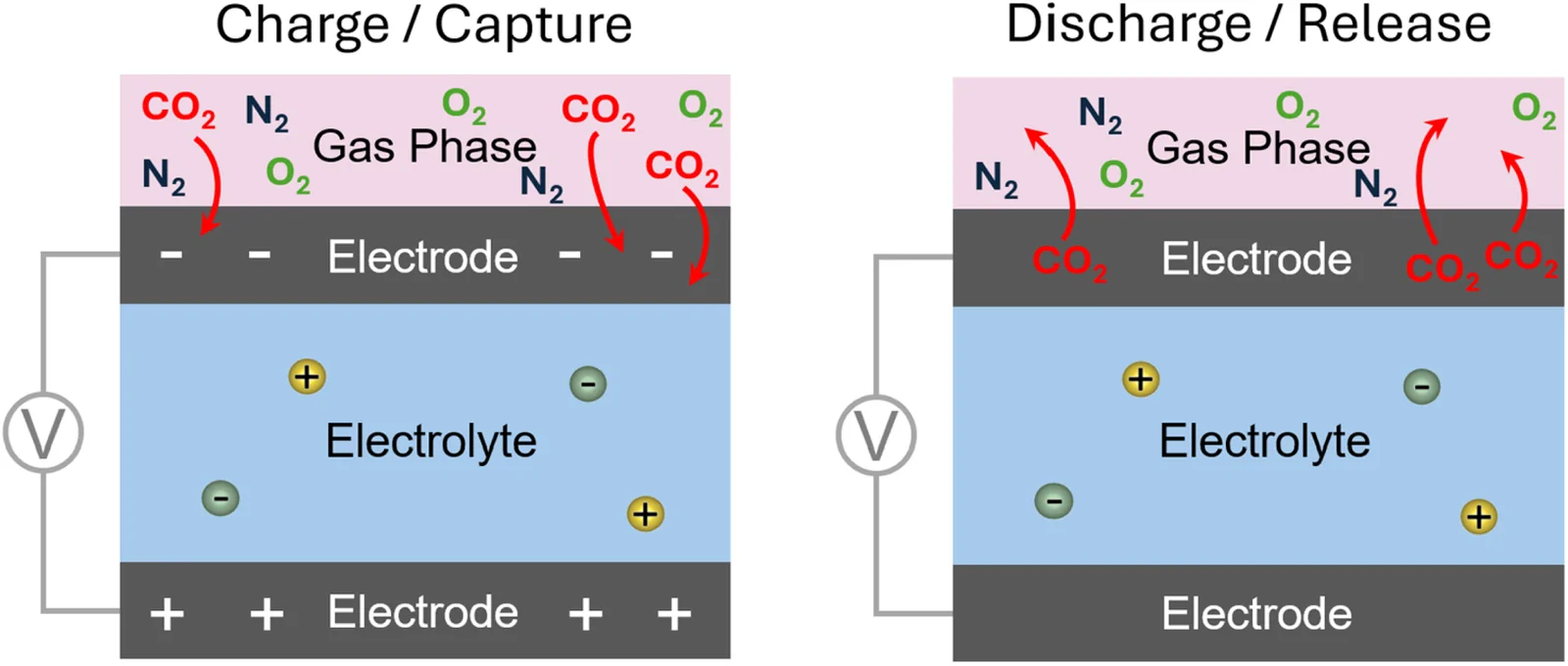
Schematic of supercapacitive swing adsorption (SSA), which uses the charging and discharging of a supercapacitor with aqueous electrolyte to reversibly and selectively capture CO_2_.

Carbon dioxide capture at an industrial scale currently relies on thermal swing processes, where CO_2_ capture and release are governed by temperature changes.^5^ Electrochemical swing methods are recently developed alternatives where CO_2_ capture and release are instead governed by a change in potential across an electrochemical cell. Electrochemical approaches may achieve higher energy efficiencies, and therefore lower costs, than thermal swing processes, as they are not constrained by the Carnot efficiency associated with heat engines. They also offer advantages in scalability and compatibility with renewable energy sources.^6,7^ Among emerging electrochemical swing approaches, Supercapacitive Swing Adsorption (SSA) is a particularly compelling candidate. First introduced in 2014 by the Landskron group,^8^ SSA reversibly and selectively captures CO_2_ using the charging and discharging cycles of aqueous supercapacitors with a gas-exposed electrode, as schematically shown in Fig. 1.

This electrochemical capture mechanism requires no costly thermal regeneration, has a long cycle lifetime, and is inherently tolerant to humidity in the gas stream. Crucially, CO_2_ is captured during charging and released during discharge. As supercapacitors are energy storage devices which exhibit high coulombic and energy efficiencies, a substantial fraction of the electrical energy input during charging can be recovered during discharge, improving overall energy efficiency of the capture process. SSA modules also make use of abundant, non-toxic, and sustainable materials. The electrodes can be fabricated from activated carbon derived from low-cost or waste biomass, and the electrolyte may range from simple salt solutions to even seawater.^9,10^ In principle, this capture technology satisfies many of the criteria that remain difficult to achieve with other approaches.

However, a critical challenge remains: SSA's CO_2_ adsorption capacity and rate are much lower than conventional systems, such as amine scrubbing.^11,12^ Reported adsorption capacities for SSA systems have increased from approximately 40 mmol per kg of active electrode material in 2014 ^8^ to around 100–250 mmol kg^−1^ in more recent studies,^13^ while amines have reported capacities of 700–2700 mmol L^−1^.^14,15^ Isolated reports demonstrate adsorption capacities of up to 500–700 mmol,^16,17^ although these systems often exhibit lower energetic and coulombic efficiencies, unclear long-term stability, and slower charging rates compared to SSA operated within more stable conditions. Similarly, most reported adsorption rates for SSA modules remain between 100–500 mmol kg^−1^ h^−1^,^13^ which compares unfavourably to the rates of 1500–5500 mmol L^−1^ h^−1^ reported for amine sorbents.^14^

Addressing this performance gap has become a central focus of the field, driving a significant number of studies to optimise electrolyte concentration^18^ and composition,^9^ carbon electrodes,^10,19,20^ charging protocols as well as voltage windows,^21,22^ gas feed composition,^16^ and scaling of devices.^23^

Despite this progress and resulting improvements in performance, the underlying capture mechanism of SSA remains unresolved. It is still unclear, at a fundamental level, what drives CO_2_ adsorption in supercapacitors, what is stored, where, and why. The field's own literature reflects this lingering uncertainty, and the undertone across SSA research, even if often tucked away as a side note or in a brief outlook section, sounds familiar:

• “Further studies are required to understand the mechanism of SSA phenomena…”^21^

• “Currently, the mechanism is based on hypotheses and reasoning, with only a limited number of experimental studies conducted.”^24^

• “A more detailed understanding of the precise mechanism of SSA would be desirable…”^13^

• “…additional investigations are necessary to fully resolve the mechanistic complexities in this field.”^25^

• *…*

As a result, the current trajectory of SSA research could be described as resembling the parable of the blind men and the elephant, in which different observers each perceive only one part of an elephant and therefore arrive at different and sometimes seemingly incompatible descriptions of the same animal. A similar pattern appears in SSA research: one study grasps the role of activated carbon, another the effect of voltage windows, and a third the influence of electrolyte composition. Each reveals an important aspect of the system's behaviour, but without a unifying theory of how SSA works at a fundamental level, these pieces do not yet connect into a coherent whole.

**Fig. 2:**
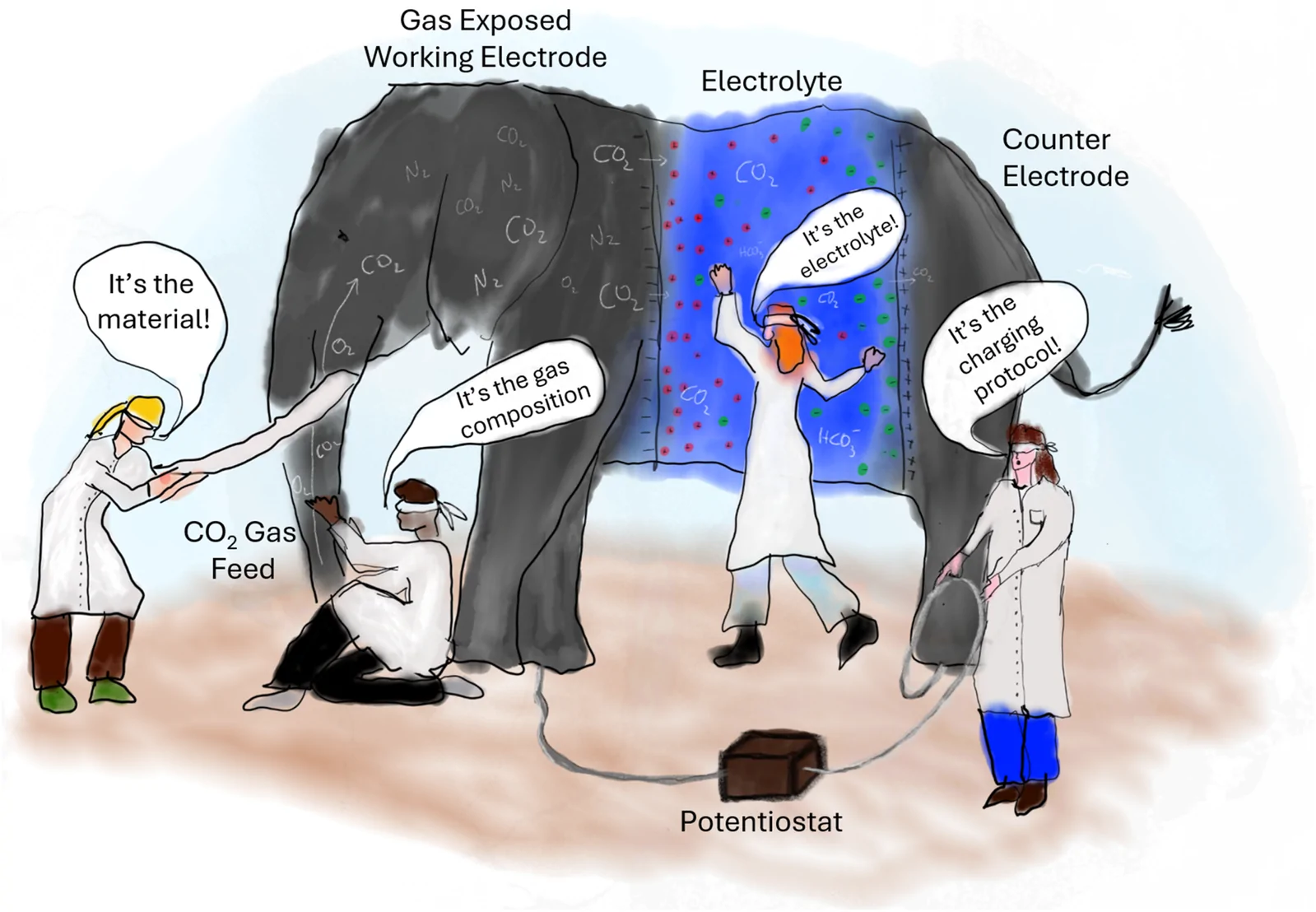
The SSA elephant in the room: Research has made rapid performance progress by optimising individual components, while important aspects of the underlying capture mechanism are still not clear. As in the parable of the blind men and the elephant, each study touches part of the system, but without understanding how SSA actually works, these pieces do not yet form a coherent whole.

This is not to diminish the progress made. These studies have significantly expanded our understanding of SSA's operating parameters and performance landscape. As illustrated in Fig. 2, proposals for improving CO_2_ capture in supercapacitors, such as “It's the material!“, “It's the electrolyte!“, or “It's the charging protocol!“, are indeed all true and important. And it is just as likely that parameters not yet identified will prove critical as well. However, these claims remain incomplete until they can be extended to say: “It's this parameter, because SSA captures CO_2_ through this mechanism”. Only then can truly targeted and predictive optimisation begin.

In this perspective, we therefore argue that the next breakthroughs in SSA will not come from further incremental tweaks, but from a shift in focus: from asking *what* works to understanding *why* it works. To guide this shift, we begin by mapping the current empirical landscape to establish what is known. We then examine the major uncertainties and conflicting results that remain, and identify experimental observations that any mechanistic framework should be able to explain. Finally, we propose possible approaches for developing such a mechanistic understanding. By clarifying where understanding is well established, where it remains fragmented, and how it might be advanced, we aim to support the progression of SSA from a scientifically interesting phenomenon and promising technique to a mature and scalable climate solution.

## What we know – mapping the current empirical landscape

**Fig. 3:**
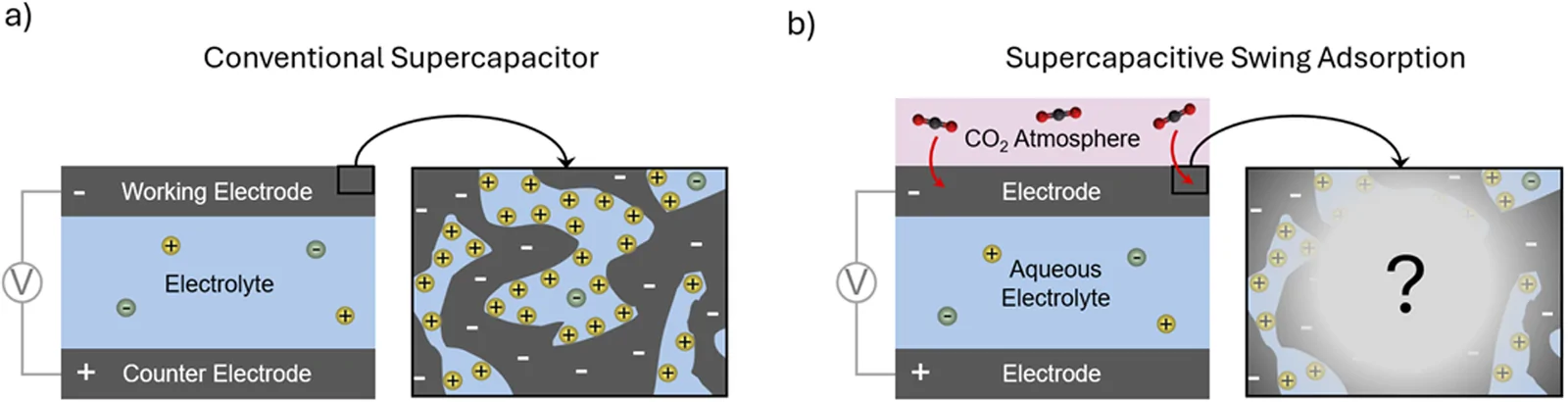
Schematic comparison of (a) a conventional supercapacitor and electric double layer (EDL) formation at the electrode–electrolyte interface and (b) a supercapacitor used for carbon capture, highlighting the unresolved underlying mechanism.

Supercapacitors are electrochemical energy storage devices in which charge is primarily stored through the formation of an electrical double layer at the electrode–electrolyte interface, without reliance on bulk faradaic reactions^26^ (Fig. 3a). In 2014, the Landskron group first reported that supercapacitors with aqueous electrolytes can also reversibly capture and release CO_2_ during charging and discharging, respectively, when one electrode is exposed to a CO_2_ containing gas phase (Fig. 3b).^8^ The underlying mechanism of this Supercapacitive Swing Adsorption (SSA) is not yet resolved, but the core phenomenon of electrochemically driven, reversible CO_2_ capture has been repeatedly confirmed, and efforts to improve performance have focused on a range of design and operational parameters.

Since its initial observation, SSA has typically been implemented using a supercapacitor composed of activated carbon electrodes, an aqueous electrolyte, and a CO_2_-containing gas stream or static CO_2_-containing headspace in contact with one electrode. Most of what is currently established in SSA research is functional in nature: which changes tend to increase adsorption capacity, and which configurations influence adsorption rate, energy consumption, time-energy efficiency and other performance metrics introduced in ref. 21.

In the absence of a well-understood adsorption mechanism, it is also not obvious which performance metric is most appropriate for SSA systems. Adsorption capacity is often normalised to the mass of the gas-exposed electrode, although one could equally argue for normalisation to electrolyte mass, total system mass, electrode area, stack volume or other quantities, since it is not yet clear which component fundamentally governs CO_2_ uptake. Normalising to electrode mass nevertheless enables rough comparison with other carbon capture technologies and has therefore become common practice. Metrics such as energy consumption per mole of captured CO_2_ or capture rate may already provide a more meaningful basis for comparison, but adsorption capacity is used here for consistency with the existing literature. In addition, direct comparison of reported performance metrics between studies remains challenging, as no standardised SSA cell architecture or measurement protocol currently exists, and even conventional supercapacitor systems often exhibit substantial variability in reported electrochemical performance despite the use of similar materials and cell configurations. As the mechanism of SSA becomes clearer, it may become necessary to reconsider how performance is defined and reported more generally.

Below, we summarise the empirical trends that have emerged across four primary domains (electrolytes, electrode materials, charging protocols, and gas composition) not as a comprehensive review, but as a frame of reference. These observations provide the foundation for the more conceptual discussion that follows as a basis for identifying where patterns break down, where results appear contradictory, and where the absence of mechanistic understanding becomes limiting. For more detailed surveys of SSA performance, we refer the reader to two recent review articles.^13,24^

### Charging protocol and voltage window

**Fig. 4:**
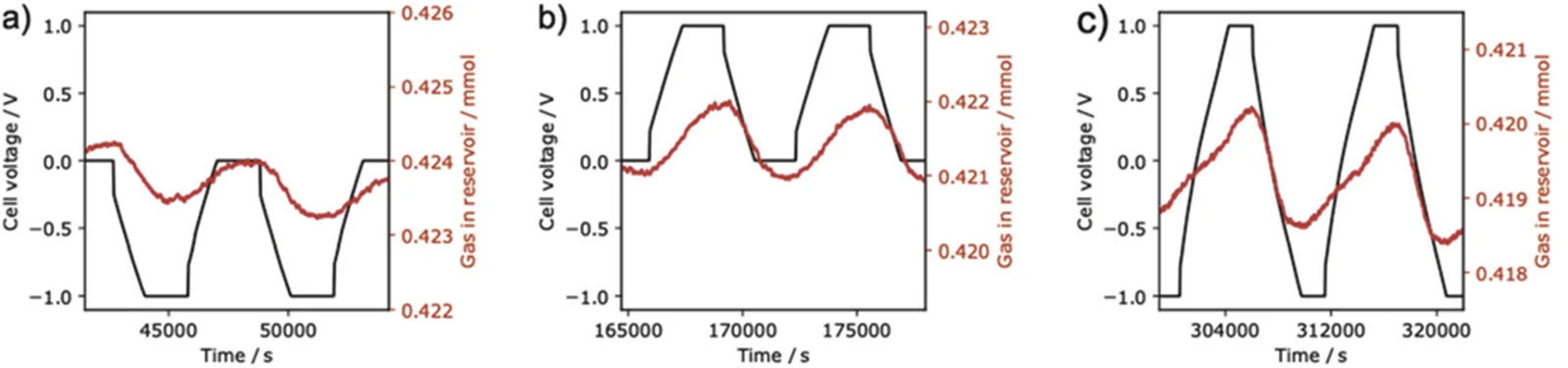
Charging protocols in SSA with (a) negative charging, associated with CO_2_ capture, (b) positive charging, associated with CO_2_ release and (c) polarity switching. Reprinted from ref. 22 with permission from the Royal Society of Chemistry (RSC), copyright 2022.

Cell charging protocols play a central role in controlling CO_2_ capture and release in SSA, and several modes of operation have been explored (Fig. 4). In the so-called “negative charging” mode, where the gas-exposed electrode is negatively polarised as the cell is charged, CO_2_ is typically captured and released again as the cell is discharged. Conversely, in the “positive charging” mode, where the gas-facing electrode is positively polarised, CO_2_ is released during charging and re-adsorbed upon discharge. Based on this observation, a “switching protocol” has also been tested, in which the polarity of the gas-exposed electrode alternates between cycles. Across the tested charging protocols, the observed behaviour suggests that adsorption is governed not by absolute polarity but by the direction of the potential change at the gas-exposed interface.^22^

The rate of charging also affects SSA performance. In symmetric cells with two carbon electrodes, CO_2_ uptake typically shows a maximum near 30 mA g^−1^, with lower and higher current densities both resulting in decreased adsorption capacities.^19,27^ However, this optimum is not universal and varies with the carbon diffusion characteristics and with the choice of counter electrode. For example, when the counter electrode is replaced with a non-carbon material, such as zinc, or a carbon with different diffusive properties, the current density dependence can shift.^27^ Related work has shown that holding the voltage for extended periods can lead to CO_2_ release, implying transient capture behaviour and offering a possible explanation for low CO_2_ uptake at slow charging rates.^25^ To address performance losses at high charging rates, some studies have introduced holding steps after charging and discharging, either at constant voltage or under open-circuit conditions. These allow additional time for equilibration and can improve CO_2_ capacity and electron efficiency, although they increase cycle time and energy losses through self-discharge.^21^

Alongside such protocol adjustments, the choice of voltage window is another investigated parameter. Increasing the window from −1.0 V to −1.4 V has been shown to significantly improve CO_2_ adsorption capacity. However, extending beyond −1.4 V leads to growing contributions from parasitic electrochemical reactions, reduced coulombic and energy efficiency, and a loss of purely capacitive behaviour.^17^ The practical limit for SSA operation in aqueous systems is therefore estimated to be about 1.4 V for CO_2_ and N_2_ mixtures, although longer-term cycling will be required to confirm stability at this voltage. The influence of gas composition on the optimal stable voltage window will be addressed in the following subsection on Gas Feed Composition.

In summary, charging protocol, current density, and voltage window all have a considerable impact on SSA behaviour. In the investigated systems, CO_2_ is captured when the gas-exposed electrode is swept to more negative potentials and released when it is swept to more positive potentials. A maximum in adsorption capacity is typically observed at an intermediate current density, with prolonged voltage holds leading to rerelease of captured CO_2_.

### Electrolyte composition and concentration

SSA has been demonstrated with a range of electrolytes, including aqueous solutions of NaCl, LiCl, KCl, MgCl_2_, NaF, NaBr, NaHCO_3_, MgBr_2_ and Na_2_SO_4_, as well as natural seawater and deionised water.^9^ Across these variations, no consistent trend has emerged linking ion identity to CO_2_ adsorption capacity, and SSA performance appears to be relatively insensitive to the specific identity of the ions in the aqueous electrolyte.^9,18,28^

**Fig. 5:**
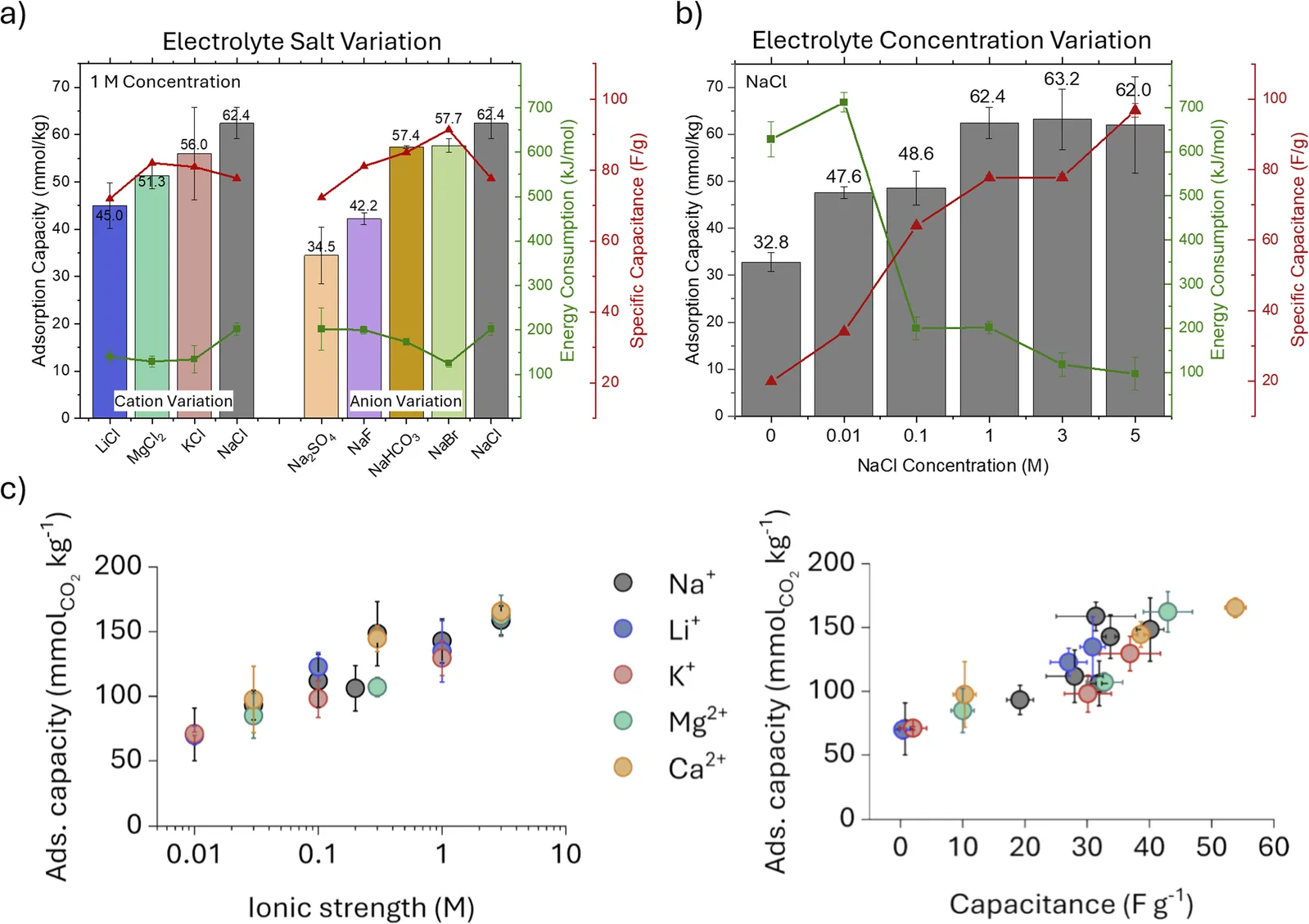
Influence of electrolyte composition and concentration on CO_2_ adsorption. (a) CO_2_ adsorption capacity, energy consumption, and specific capacitance for different electrolyte salts at a concentration of 1 M, plotted from data reported in ref. 9. (b) Effect of NaCl concentration on CO_2_ adsorption capacity, energy consumption, and specific capacitance, plotted from data reported in ref. 18. (c) CO_2_ adsorption capacity as a function of ionic and d) relationship between CO_2_ adsorption capacity and specific capacitance strength for various electrolyte ions, Reprinted from ref. 28 with permission from the American Association for the Advancement of Science (AAAS), copyright 2026.

As shown in Fig. 5a), at a 1 M concentration, varying the cation relative to NaCl produced the following order of CO_2_ adsorption capacity: LiCl < MgCl_2_ < KCl < NaCl, with a maximum variation of 39%. Varying the anion produced a broader variation in performance, with adsorption capacities differing by as much as 81% across the sequence: Na_2_SO_4_ < NaF < NaHCO_3_ ≈ NaBr < NaCl.^9^ Within that salt-variation study, the observed performance trends did not correlate with the basicity of the solution nor the solubility of CO_2_, or other readily identifiable physicochemical electrolyte properties.^9^ By contrast, however, recent work points to a dependence of adsorption capacity on the initial electrolyte pH, with more basic solutions exhibiting greater uptake, suggesting that pH may play an important role.^28,29^

Rather than the ion identity itself, the concentration was found to be far more determining of the SSA performance. For example, concentration effects studied using NaCl showed CO_2_ adsorption capacities increasing between 0 and 1 M, but little further improvement at higher concentrations (Fig. 5b)).^18^ Similarly, other recent work demonstrated that increasing electrolyte concentration improves capture performance across many different ions (Fig. 5c), and that the trend correlates more closely with the energy storage capacitance of the supercapacitor than with the specific chemical identity of the ions (Fig. 5d).^28^ Interestingly, SSA has also been demonstrated in deionised water, where no initial ions are present in solution, and the adsorption capacity was comparable to that observed in low concentration electrolyte solutions, although the energy consumption was substantially higher due to the much greater internal resistance of the deionised water cell.^18^

Overall, SSA behaviour appears broadly similar across a wide range of aqueous electrolyte systems, as is also observed for conventional aqueous supercapacitors. While the specific identity of the electrolyte ions could influence performance to some extent, it does not appear to be the dominant factor, and no clear trends have yet emerged. Instead, ionic strength appears to be the more important parameter, with solution pH potentially also contributing.

### Electrode material

**Fig. 6:**
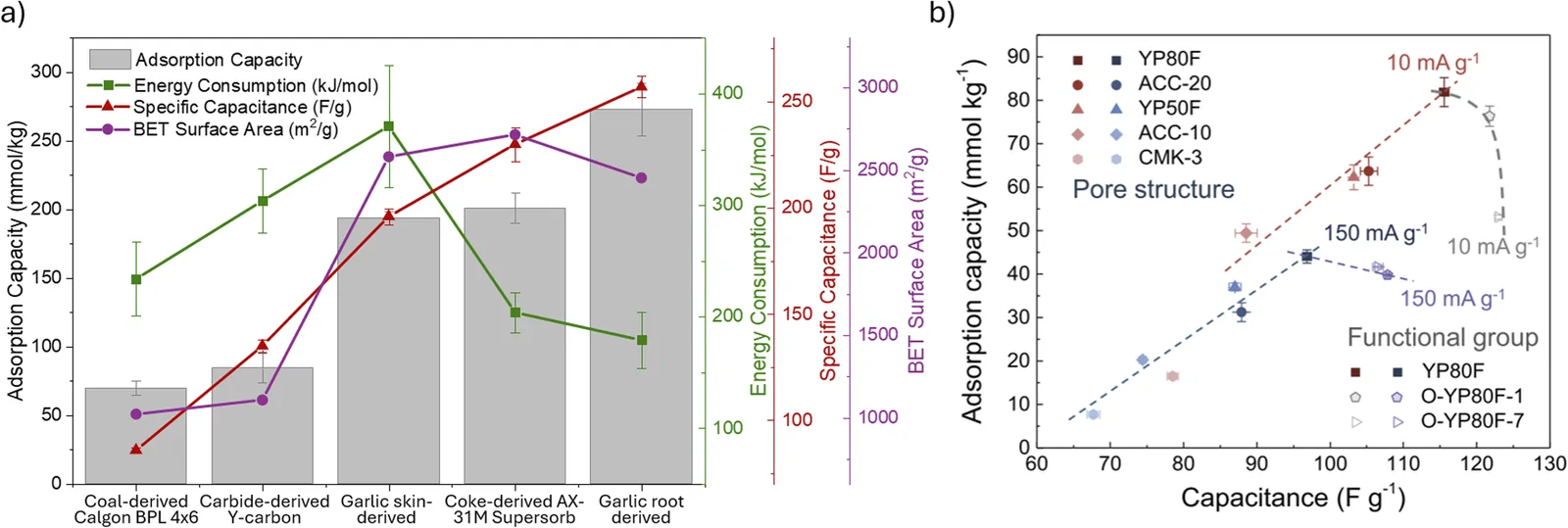
(a) CO_2_ adsorption capacity, energy consumption, specific capacitance, and BET surface area for different activated carbons, plotted using data reported in ref. 10. (b) Correlation between capacitance and CO_2_ adsorption capacity (at 10 and 150 mA g^−1^) for various activated carbon electrodes, including the effects of surface functionalisation through oxidation treatments. Reprinted from ref. 19 with permission from Springer Nature, copyright 2024.

SSA performance is also influenced by the choice of carbon electrode, although no single structural or compositional parameter has yet emerged as a reliable predictor of adsorption behaviour.^10,19,20^ A broadly positive correlation has been observed between gravimetric capacitance and CO_2_ adsorption capacity: carbons with higher specific capacitance in conventional supercapacitor applications often also exhibit higher SSA capacities (Fig. 6), in line with the general trend observed across different electrolytes (Fig. 5). For instance, as shown in Fig. 6a), the commercial activated carbon BPL 4 × 6 (specific capacitance 80 F g^−1^) captures around 70 mmol kg^−1^, whereas a custom biomass-derived carbon from garlic root (257 F g^−1^) was shown to achieve 273 mmol kg^−1^, a nearly fourfold increase in CO_2_ capacity.^10^

However, this trend is not universal. Carbons with similar capacitance can exhibit markedly different CO_2_ adsorption capacities, while materials with substantially different capacitances may display comparable CO_2_ uptake. For example, garlic skin-derived and coke-derived carbons exhibit very similar CO_2_ adsorption capacities, despite different capacitance values (Fig. 6a)). Likewise, a carbon with a predominantly mesoporous structure achieved comparable specific capacitance but showed significantly lower CO_2_ adsorption capacities, particularly at slow charging rates (Fig. 6b)), light red data circle at 10 mA g^−1^ for CMK-3).^19^ More notably, the introduction of oxygen functional groups has been shown to increase capacitance while reducing CO_2_ uptake (Fig. 6b), grey and purple data points for oxidised YP80F), demonstrating that higher capacitance does not necessarily translate into higher SSA performance and can even produce the opposite trend.^19^ These observations suggest that capacitance is a useful empirical indicator of SSA performance, but not a determining material parameter.

More fundamentally, even relating capacitance to specific structural properties in porous activated carbons is difficult, as these materials are complex and often poorly defined. This is illustrated in Fig. 6a) where garlic-derived, coke-derived and garlic root derived-carbons exhibit similar BET surface areas, but progressively increasing capacitance values. Changes in precursor composition, activation conditions, or post-treatment typically influence multiple properties simultaneously, including pore size distribution, surface chemistry, wettability, electrical conductivity, and structural disorder. As a result, it is difficult to vary individual material parameters independently and to establish clear structure–function relationships.

In the absence of a known capture mechanism, this challenge becomes even greater for SSA, as it is difficult to empirically determine which structural properties are truly beneficial for CO_2_ adsorption. Fig. 6a illustrates this ambiguity, as the CO_2_ adsorption capacity appears, in some cases, to follow BET surface area and, in others, capacitance, without showing a consistent relationship with either parameter. It might be assumed that pore sizes favourable for high capacitance would also be beneficial for SSA performance. However, as discussed above, carbons with predominantly mesoporous structures can exhibit high capacitance but poor SSA performance,^19^ indicating that a clear optimal pore size distribution for SSA has not yet been established. In conventional supercapacitors, higher capacitance is often associated with increased structural disorder^30^ rather than with a single well-defined pore size. Whether structural disorder is similarly beneficial for SSA performance remains unclear. More recently, pore network tortuosity has been identified as a key parameter controlling rate capability in porous carbon supercapacitors.^31^ Carbons that demonstrate slower charging behaviour in supercapacitors have likewise been reported to exhibit slower CO_2_ adsorption kinetics,^25^ suggesting a possible link between SSA kinetics and pore network tortuosity.

Compared to structural properties, surface chemistry has received comparatively little attention in the context of SSA. Functional groups and heteroatom doping are known to improve capacitance, wettability, and electrolyte accessibility in conventional supercapacitor applications,^32,33^ and carbons with improved wettability and pore filling have generally shown better SSA performance.^20,24^ However, systematic studies linking surface functional groups directly to CO_2_ adsorption behaviour remain scarce. One of the few reported examples, also discussed above, showed that oxidation of the carbon surface increased capacitance but reduced CO_2_ uptake (Fig. 6 b)),^19^ indicating that surface chemistry can influence electrochemical and adsorption behaviour in different and potentially competing ways. Beyond such isolated observations, the role of surface chemistry in SSA remains largely unexplored.

In sum, while the choice of carbon clearly modulates SSA performance, no generalisable structure–function relationship has yet emerged, besides a mostly positive correlation between specific capacitance and CO_2_ adsorption capacity.

### Gas feed composition

SSA behaviour has been examined under a range of gas compositions, using both static headspace experiments and continuous-flow systems. In static experiments, the gas phase typically consists of pure CO_2_, O_2_, or N_2_ contained within a sealed headspace, with pressure changes serving as a direct measure of changes in the amount of gas-phase species. Flow-based experiments more commonly use variable feed mixtures or simulated flue gases, such as 15% CO_2_ and 85% N_2_, where the outlet composition is monitored using CO_2_ and O_2_ sensors.

**Fig. 7:**
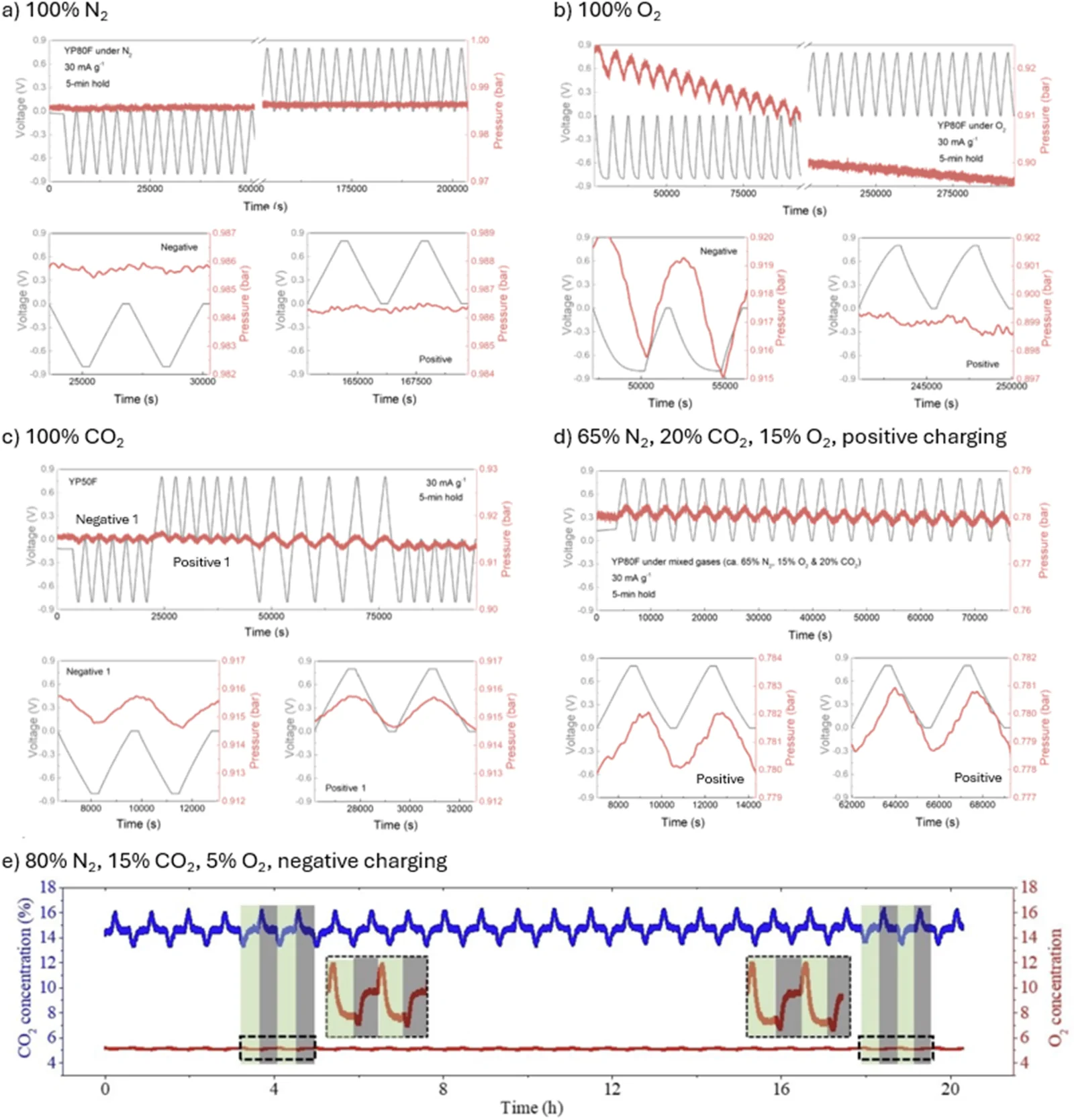
SSA behaviour of different gases and mixtures. (a–d) Static headspace measurements showing pressure responses during charge–discharge cycling under (a) 100% N_2_, (b) 100% O_2_, (c) 100% CO_2_, and (d) a mixed gas atmosphere (approximately 65% N_2_, 20% CO_2_, 15% O_2_). The enlarged panels show individual adsorption–desorption cycles during negative and positive charging for (a–c), while only positive charging was performed in (d). Reprinted from ref. 19 with permission from Springer Nature, copyright 2024. (e) Flow cell measurements under simulated flue gas conditions (80% N_2_, 15% CO_2_, 5% O_2_) during negative charging, showing CO_2_ and O_2_ concentration changes during cycling. Reprinted from ref. 16 with permission from Wiley-VCH GmbH, copyright 2024.

Across these studies, N_2_ consistently shows no measurable SSA response during either positive or negative charging (Fig. 7a)).^19^ In contrast, during negative charging, pure O_2_ exhibits largely reversible uptake, although some irreversible uptake has been observed. During positive charging, pure oxygen shows almost no SSA activity (Fig. 7b).^19^ For comparison, results for pure CO_2_ are shown in Fig. 7c), while Fig. 7d) shows a CO_2_/N_2_/O_2_ gas mixture during positive charging only, as the negligible activity of N_2_ and O_2_ enables interpretation from pressure measurements in static headspace experiments. Fig. 7e) shows an example of simulated flue gas measurements during negative charging in a flow cell system, which allows direct monitoring of gas concentrations. Consistent with the static headspace experiments of pure O_2_, predominantly reversible SSA activity was also observed in the gas mixtures and increased irreversible oxygen uptake was mainly reported when the cell voltage window exceeded 0.6 V.^16^ Interestingly, in this simulated flue gas mixture, reversible oxygen uptake during negative charging occurs in the opposite phase to CO_2_ behaviour, meaning oxygen is released during CO_2_ adsorption and adsorbed again during CO_2_ release (Fig. 7e)).^16^

Beyond flue-gas-relevant compositions, SSA has also been demonstrated to operate effectively at low CO_2_ concentrations relevant to direct air capture in a preprint study.^34^ In this study, at 400 ppm CO_2_, the measured adsorption capacity is only around six times lower than at 15% CO_2_, despite the CO_2_ partial pressure being approximately 375 times smaller, indicating a strongly non-linear dependence on CO_2_ concentration in the gas phase.

Owing to its use of aqueous electrolytes, SSA is also inherently insensitive to moisture in the gas stream, in contrast to many other CO_2_ capture technologies. Additionally, oxygen, which is a well-known cause of degradation in many carbon capture systems, has been shown to be tolerated in SSA.^16^ In mixed gas conditions of 20% CO_2_, 15% O_2_ and 65% N_2_, stable cycling for at least 1000 cycles has been demonstrated, with coulombic efficiencies exceeding 99.8%.^19^

Beyond just oxygen tolerance, the presence of oxygen was demonstrated to even be beneficial and enhance CO_2_ capture behaviour in some cases.^16^ Replacing a simulated flue gas mixture of 15/85% CO_2_/N_2_ with 15/80/5% CO_2_/N_2_/O_2_ has been shown to increase CO_2_ adsorption by up to elevenfold with 3 M MgBr_2_ at 0.6 V (∼750 mmol kg^−1^), and by four to fivefold with 3 M MgCl_2_ at 1 V (∼320 mmol kg^−1^). Further increasing the oxygen concentration (10%, 15%, 20%) resulted in similar enhancements.^16^ For MgBr_2_, voltages above 0.6 V introduced a trade-off between higher adsorption capacity and reduced coulombic efficiency, along with some irreversible oxygen uptake by the carbon electrodes.^16^ The mechanistic basis for the oxygen-assisted enhancement remains not yet understood.

In summary, SSA shows excellent selectivity for CO_2_ over N_2_ with stable performance across a wide range of CO_2_ concentrations in the feed gas. O_2_, however, appears to play a more complex role, with studies reporting both reversible and irreversible uptake behaviour as well as, under certain conditions, enhanced CO_2_ capture performance. At the same time, oxygen exposure may also contribute to electrode degradation and decreased coulombic efficiencies and cycle stabilities, and its precise role in SSA remains unclear.

## What we do not know (yet) – unresolved mechanism and open questions

Despite a growing body of research and continued improvements in reported performance, the underlying mechanisms and driving forces by which charging of a supercapacitor leads to CO_2_ capture and release remain unresolved. Mechanistic ideas have been proposed, yet no study has unambiguously confirmed a capture pathway or established how multiple processes might coexist or compete during operation. In the following, we introduce the mechanistic ideas proposed to date and assess their compatibility with the available experimental evidence. Beyond this comparison, we more broadly outline the experimental trends and constraints imposed by current results that we believe any viable mechanistic proposal for SSA, whether existing or future, should account for.

### Proposed mechanisms

**Fig. 8:**
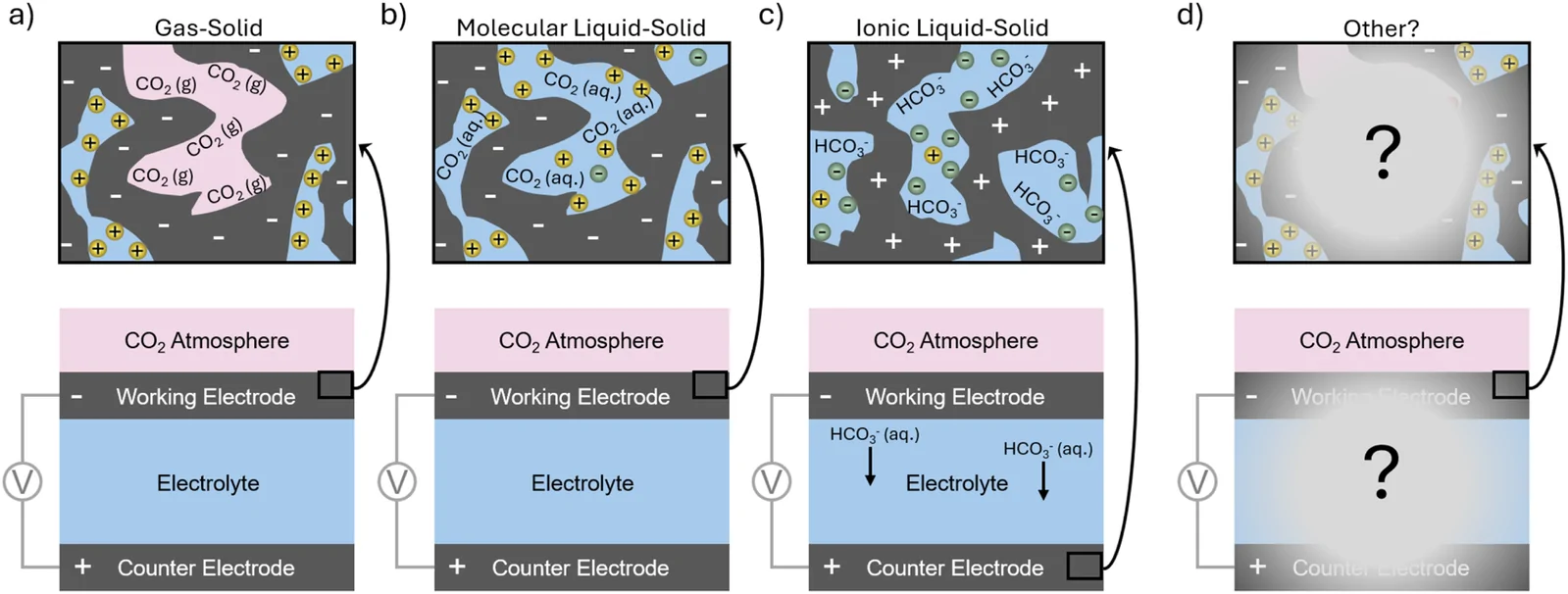
Current ideas proposed to explain how SSA might operate, first put forward in ref. 18, (a) gas–solid mechanism, (b) molecular liquid–solid mechanism and (c) ionic liquid–solid mechanism. Panel (d) highlights potential alternative pathways, such as pH swing, or other mechanisms not yet identified.

So far, three main mechanisms have been proposed to explain CO_2_ capture in SSA,^18^ as illustrated in Fig. 8: (a) the gas–solid mechanism, (b) the molecular liquid–solid mechanism and (c) the ionic liquid–solid mechanism. Within SSA literature, these mechanisms are generally treated as working hypotheses rather than a complete mechanistic picture, and SSA may in practice operate through a combination of these processes, or *via* an alternative mechanism that has yet to be identified (Fig. 8d)).

The gas–solid mechanism (Fig. 8a) assumes that gaseous CO_2_ molecules adsorb into pores that remain unwetted by electrolyte. It is further assumed that formation of the electric double layer (EDL) in adjacent wetted pores shifts the Fermi level, thereby altering the adsorptivity of the neighbouring gas-filled pores for CO_2_, which drives capture and release.

In the molecular liquid–solid mechanism (Fig. 8b) dissolved molecular (neutral) CO_2_ is assumed to accumulate at the electrode–electrolyte interface, owing to a hypothesised difference in solubility between the EDL region and the bulk electrolyte.

The ionic liquid–solid mechanism (Fig. 8c) assumes that CO_2_-derived ionic species, most prominently bicarbonate (HCO_3_^−^), is electrostatically repelled from the negatively polarised electrode due to like–charge interactions, and attracted towards the positively polarised electrode. As a consequence, the electrolyte in contact with the negative electrode becomes depleted in bicarbonate species, thereby driving additional CO_2_ uptake from the gas phase to restore equilibrium.^18^

More recent studies have also pointed towards an electrochemical pH swing as a potential mechanism for CO_2_ capture in supercapacitor systems.^28,35^ Here, it is assumed that charging induces pH changes at the electrodes, which shift the dissolved inorganic carbon equilibrium and thereby drive exchange with the gas phase.^13^

The ionic liquid–solid mechanism (Fig. 8c) has more recently also been extended to include proton adsorption at the negatively polarised electrode, which may induce local pH changes and thereby drive CO_2_ uptake.^13^ However, in the established definition of the ionic liquid–solid mechanism, the primary driving force for CO_2_ capture is electrostatic redistribution and depletion of (bi)carbonate ions. In contrast, the pH-swing-based interpretation (grouped as “Other”, Fig. 8d) treats the pH-dependent shift in dissolved inorganic carbon equilibria as the primary driving force for CO_2_ capture. Accordingly, the pH-swing-based mechanism is treated separately from the ionic liquid–solid mechanism because the two mechanisms are governed by different dominant driving forces, and because pH changes in supercapacitors may arise from processes including, but not limited to, proton adsorption within the electric double layer.

Having briefly introduced these mechanisms, which differ in the driving forces and carbon speciation they invoke, we now turn to a set of recurring experimental observations that provide constraints against which existing and future mechanistic proposals can be assessed.

### Polarity dependence and transient capture behaviour

Arguably, one of the most informative constraints provided by existing experiments is the pronounced polarity dependence of SSA, with CO_2_ uptake consistently associated with negative charging and release upon discharging or positive charging of the gas-exposed electrode (Fig. 4). While such polarity dependence does not exclude mechanisms involving neutral species, such as the gas–solid or the molecular liquid–solid mechanism, it is more naturally explained by the participation of charged species or by equilibria that are coupled to interfacial charge. These include ionic liquid–solid or pH swing mechanisms, both of which inherently encode polarity sensitivity.

Closely related to polarity dependence, CO_2_ capture has also been shown to be transient, with long voltage holds or slow charging leading to the release of previously captured CO_2_, as outlined earlier. This indicates competing processes at the two electrodes, where conditions that favour CO_2_ uptake at one electrode simultaneously favour release at the counter electrode or gradual equilibration between the two electrodes.

In both ionic liquid–solid and pH-swing-based mechanisms, transient capture behaviour can arise naturally from competing processes at the two electrodes, as well as from gradual re-equilibration during extended voltage holds. In the ionic liquid–solid mechanism, depletion of (bi)carbonate species at one electrode drives additional CO_2_ uptake from the gas phase, while accumulation at the counter electrode may simultaneously favour CO_2_ release. Similarly, in a pH-swing-based mechanism, one electrode may become increasingly basic and favour CO_2_ capture, while acidification at the other electrode promotes CO_2_ release. Over extended voltage holds, these gradients may also progressively relax through diffusion and equilibration processes, reducing the net driving force for continued CO_2_ uptake.

Supporting this interpretation, it was shown that replacing the counter electrode with a non-porous zinc electrode suppresses the loss of adsorption capacity under slow charging conditions.^25^ Consistent with this picture, finite element simulations of a model SSA system have predicted contrary (bi)carbonate ion concentration changes at the electrodes, in line with the polarity sensitivity and transient capture behaviour expected for ionic liquid–solid mechanisms.^27^ Similarly, experimental studies have shown that in a conventional supercapacitor, pH changes of opposite sign develop at each electrode during operation, with pH increases during negative charging and pH decreases upon positive polarisation,^36^ which could be further enhanced in supercapacitors by field-enhanced water dissociation processes such as the second Wien effect.^37^ A recent study further reported that, after disassembling an SSA module and titrating the electrolyte, elevated alkalinity and the presence of a weak base-strong acid titration curve were observed at the negative electrode, evidencing the presence of (bi)carbonate species.^35^ Therefore, both ionic liquid–solid and pH swing capture remain highly compatible with the observed polarity dependence and transient capture behaviour.

### Capture kinetics

In addition to the transient capture behaviour observed at slow charging rates, a decrease in CO_2_ uptake has also been reported at higher charging rates. CO_2_ capture in SSA systems generally occurs on timescales that are slower than would be expected for physisorption alone,^13^ which suggests that a purely gas–solid mechanism is unlikely to account for the observed behaviour. With increasing charging rate, the electrochemical capacitance of supercapacitors decreases, and carbons with poorer rate capability have correspondingly been reported to exhibit slower CO_2_ adsorption kinetics.^25^ The reduction in CO_2_ adsorption capacity, however, is substantially larger than the corresponding decrease in capacitance and therefore cannot be explained by changes in charge storage alone.^21^ Instead, this behaviour points towards additional rate-limiting processes, such as diffusion, gas–liquid exchange, or equilibration of dissolved carbon species, as would be consistent with molecular or ionic liquid–solid, or pH-swing-based mechanisms.

### Role of CO_2_ in the electric double layer

It has been reported that the addition of CO_2_ can increase the specific capacitance of supercapacitors with porous carbon electrodes, both in ionic liquid-based electrolytes^38^ and in ultrahigh-concentration aqueous electrolytes.^39^ It has been suggested that CO_2_ may induce an ion-bridging effect, potentially reducing resistance or enabling more efficient packing within the electric double layer.^38^ In the case of ultrahigh-concentration aqueous electrolytes (5 M NaClO_4_), the electrolyte pH decreased upon CO_2_ addition as expected; however, this effect was controlled by adjusting the pH of the CO_2_-free electrolyte using sulfuric acid. The observed capacitance increase therefore could not be attributed solely to pH changes. Similarly, the addition of Na_2_CO_3_ or NaHCO_3_ to introduce (bi)carbonate ions did not lead to measurable changes in capacitance, indicating that the presence of CO_2_-derived anions alone does not account for the observed increase in capacitance.^39^ Such investigations have not yet been performed in a carbon capture context, but suggest a possible involvement of molecular CO_2_ in the EDL, which could be related to capture and would be consistent with a molecular liquid–solid mechanism.

In a context more directly related to SSA, experiments using non-porous gold electrodes in aqueous electrolytes showed that the introduction of CO_2_ decreased the capacitance at both charging polarities.^40^ This behaviour was attributed to the adsorption of molecular CO_2_ at the charged surface, leading to polarity-independent CO_2_ uptake while simultaneously reducing the effective interfacial area available for EDL formation. This is in contrast to the previously observed capacitance enhancement in activated carbon, but directly supports a molecular liquid–solid mechanism in the gold-electrode system. Overall, it remains unclear to what extent these findings can be translated to porous activated carbon electrodes, and whether a molecular CO_2_ capture mechanism plays a significant role in these SSA systems. In particular, in what way the influence of CO_2_ on the structure or properties of the EDL might be coupled to CO_2_ capture remains an open question.

### Gas composition, selectivity and oxygen enhancement

SSA has been demonstrated for a range of gas compositions and exhibits selectivity toward CO_2_ over other gas components. Such selectivity is compatible with mechanisms involving aqueous CO_2_ chemistry, including pH swing and ionic liquid–solid mechanisms, which rely on the hydrolysis of CO_2_ to bicarbonate and carbonate species, and on the perturbation of their local equilibrium by either pH or electrostatic driving forces. In contrast, gas–solid and molecular liquid–solid mechanisms would require a more specific explanation for CO_2_ selectivity, as they do not inherently distinguish CO_2_ from other small, nonpolar gas molecules like N_2_, O_2_, or noble gases. One proposed explanation is that CO_2_, acting as a Lewis acid, may interact more strongly with the increasingly electron-rich and therefore more Lewis-basic negatively polarised carbon surface, particularly with the π–electron system of the charged electrode, whereas O_2_ and N_2_ lack similarly pronounced Lewis acidic properties.^13^

In addition to selectivity, an increase in adsorption capacity upon the addition of oxygen to the gas stream has been reported.^16^ This behaviour is not readily explained by adsorption-based SSA mechanisms such as gas–solid adsorption or molecular or ionic liquid–solid interactions, which do not provide a clear role for oxygen. However, oxygen may play a role within pH-swing-based interpretations. Electrochemical oxygen reduction reactions (ORR) are known to occur on carbon electrodes and can generate hydroxide ions, which would increase the local basicity and thereby enhance CO_2_ adsorption capacity, consistent with previous observations reported in recent works.^28,29^ However, experimental observations show that oxygen is released during CO_2_ adsorption and re-adsorbed during CO_2_ release,^16^ which does not directly match simple oxygen reduction behaviour. This suggests that the role of oxygen in SSA may involve more complex electrochemical or surface reactions, leaving its influence an open mechanistic question.

Another important observation is reported in a recent preprint, which demonstrated that SSA remains effective under direct air capture conditions, where the CO_2_ concentration is approximately 0.04% (≈400 ppm). Although this concentration is about 375 times lower than that of a simulated flue gas containing 15% CO_2_, the reported adsorption capacity was only reduced by a factor of six.^34^ This observation is difficult to explain with mechanisms that are directly limited by the equilibrium concentration of dissolved CO_2_, such as gas–solid adsorption or molecular liquid–solid mechanisms, for which Henry's law would predict an approximately linear scaling of available dissolved CO_2_ with gas-phase CO_2_ partial pressure. By contrast, mechanisms involving pH-driven carbonate equilibria or electrostatically driven redistribution of (bi)carbonate species may exhibit a more complex, non-linear dependence on CO_2_ partial pressure. Particularly, in the ionic liquid–solid mechanism, electrostatically driven redistribution of (bi)carbonate species could, in principle, proceed without being directly limited by the instantaneous CO_2_ partial pressure. The observed non-linear dependence therefore does not uniquely identify a mechanism but suggests that SSA may not be governed solely by equilibrium CO_2_ dissolution.

### Electrode material and electrolyte effects

As outlined above, both the carbon electrode material and the electrolyte influence SSA performance. Beyond a general correlation between the energy storage performance of the carbon and its capture performance, no clear relationship between specific carbon properties and CO_2_ uptake has yet emerged. It therefore remains an open question which carbon characteristics govern SSA performance and how these might be predicted or optimised.

For the gas–solid mechanism to be feasible, a significant amount of unwetted pore space within the electrode is required, where CO_2_ could adsorb directly from the gas phase. However, experimental studies have consistently shown that carbons with improved wettability generally exhibit better SSA performance.^20,24^ Moreover, the long-term stability of CO_2_ capture is difficult to justify with mechanisms that rely on persistent dry pore space, given phenomena such as electrowetting in porous carbons, which would be expected to progressively reduce the volume of unwetted pore space during operation.^41^

Similarly to the carbon, no clear trend has been identified with respect to the electrolyte salt. In many cases, the identity of the electrolyte ions appears to have only a weak influence on capture performance. This is somewhat counterintuitive for the ionic liquid–solid mechanism, where (bi)carbonate ions are expected to compete with electrolyte anions for space within the EDL. In particular, uptake would be expected to be enhanced in deionised water or low-concentration electrolytes, where charge compensation relies predominantly on (bi)carbonate ions, leading to larger perturbations of the local carbonate equilibrium. Experimentally, however, the opposite trend is observed, with CO_2_ capacity increasing with increasing electrolyte salt concentration.^9^

In addition, the solubility of CO_2_ in aqueous electrolyte solutions generally decreases with increasing salt concentration due to salting-out effects,^42^ which reduce the equilibrium concentration of dissolved molecular CO_2_. This behaviour is difficult to reconcile with the molecular liquid–solid mechanism, which relies on dissolved molecular CO_2_ at the electrode surface. If SSA performance were limited by the availability of dissolved CO_2_, increasing electrolyte concentration would be expected to reduce adsorption capacity rather than increase it.

However, interpreting such trends is complicated by the difficulty of extrapolating bulk solution behaviour to confined pore environments, where local concentrations, solvation structure, and chemical equilibria may differ significantly from bulk values. Traditionally, the relatively low equilibrium concentration of bicarbonate in bulk solution has been used as an argument against ionic liquid–solid mechanisms.^12,40^ However, up to a 30-fold oversolubility of CO_2_-derived species in porous carbons has been observed, suggesting that local concentrations within pores may substantially exceed bulk equilibrium values.^43^ Nevertheless, the weak dependence of SSA on ion identity and the positive relationship between increasing electrolyte concentration and capture performance are more readily compatible with pH-swing-based interpretations, in which (bi)carbonate ions do not directly compete with electrolyte ions for adsorption sites, and capture is not limited by dissolved molecular CO_2_.

Interestingly, although the identity of the electrolyte ions generally has only a weak influence on SSA performance, as discussed above, a much more pronounced ion dependence is observed when oxygen is present in the gas stream. For example, MgBr_2_ has been reported to yield an approximately elevenfold enhancement in capture performance, compared to a roughly fourfold increase for MgCl_2_.^16^ Notably, the fourfold increase for MgCl_2_ was obtained at a higher charging voltage (1 V) compared to 0.6 V for MgBr_2_, suggesting that the electrolyte effect is particularly significant given that higher charging voltages typically lead to increased CO_2_ capture. It has been hypothesised that bromide ions may undergo electrochemical conversion to hypobromite (BrO^−^) at the negative electrode, which is a weak base and could contribute to local alkalinisation. Such behaviour would be consistent with a pH-swing-based contribution to the oxygen enhancement and would explain a more pronounced ion dependence under an oxygen atmosphere.

Overall, while existing experiments have revealed several important trends, no single material parameter or electrolyte property has yet emerged as a definitive marker for SSA performance. Identifying which carbons or electrolytes perform well, and why, remains strongly dependent on the operative capture mechanism, which itself has yet to be conclusively established.

## What we need to know – outlook and possible future directions

The preceding sections have highlighted a broad range of experimental observations that provide valuable insight into SSA performance while placing important constraints on possible capture mechanisms. At the same time, these observations expose persistent knowledge gaps and have yet to converge on a coherent explanation of how supercapacitors capture CO_2_ or what the dominant driving forces are. As a result, despite the growing body of informative experimental evidence, a lack of mechanistic understanding has emerged as the central bottleneck for further progress in SSA. Against this background, the following discussion focuses on how the constraints and gaps identified above can guide future experiments aimed at revealing the fundamental capture mechanism.

Viewed in the context of the evidence discussed here, several mechanisms remain plausible and compatible with many reported behaviours, most prominently the ionic liquid–solid and pH-swing-based interpretation. In contrast, a growing body of work has revealed inconsistencies with a purely gas–solid mechanism, leading the literature to increasingly move away from this mechanism. Nevertheless, it remains unresolved based on current research alone, to what extent CO_2_ capture in SSA proceeds *via* ionic or molecular liquid–solid, or pH-swing processes, or whether an alternative mechanism, consistent with the constraints outlined in this perspective, may be operative.

Therefore, further progress toward mechanistic understanding is needed and will likely require direct insight into the speciation and spatial distribution of CO_2_-derived species, both in the electrolyte and within the porous electrode. Techniques such as NMR spectroscopy appear particularly promising in this regard, as they can distinguish between different carbon species (CO_2_, HCO_3_^−^, CO_3_^2−^), and between in-pore and ex-pore environments and allow for quantification of their concentrations.^43^ Complementary approaches that provide more *in situ* or *operando* access to the SSA cell, for example through optical or spectroscopic windows, would further help bridge the gap between bulk measurements and interfacial processes and could help clarify the role of CO_2_ or related ions in the electric double layer. Likewise, the use of pseudocapacitive electrode materials may be informative for determining whether the observed SSA behaviour is restricted to purely capacitive processes.

In addition, new experimental configurations that allow time-resolved monitoring of electrolyte composition, speciation, or pH could provide valuable insight without relying on *ex situ* analysis or cell disassembly. Such approaches could help establish quantitative relationships between parameters such as pH changes or (bi)carbonate concentration and CO_2_ capture behaviour, thereby enabling assessment of the relative contributions of different mechanisms. To complement these efforts, atomistic modelling and other theoretical approaches are likely to play an important role in linking experimental observations.^43–45^

Beyond this, targeted experimental strategies aimed at excluding specific mechanistic pathways may be equally informative. Even if a single mechanism cannot be conclusively proven, others may be ruled out under well-defined conditions. For example, the use of buffered electrolytes could help suppress pH changes and thereby test the contribution of pH-swing-based mechanisms as compared to the ionic liquid–solid mechanism. Similarly, the use of aprotic solvents and organic electrolytes that do not form bicarbonate or related adducts could help to assess the relevance of mechanisms involving molecular CO_2_ alone. At the same time, such systems may exhibit fundamentally different behaviour due to changes in solubility, viscosity, and ion transport, which must be considered carefully.

In summary, despite the current lack of mechanistic clarity, the growing body of experimental results and the expanding range of available experimental tools provide a strong foundation for progress. The combination of advanced characterisation techniques, improved model systems, and deliberately designed mechanistic tests now offers a realistic pathway toward identifying the dominant processes governing CO_2_ capture in SSA. Ultimately, such insight would position SSA to evolve from an empirically driven approach into a predictive, rationally designed, and potentially scalable carbon capture technology.

## Author contributions

M. S.: conceptualisation, visualisation/artwork, writing – original draft, writing – review and editing, project administration; Z. C.: writing – review and editing; C. W.: writing – review and editing; S. E. W.: writing – review and editing; J. S. T.: writing – review and editing; G. E. M.: writing – review and editing; A. C. F.: conceptualization, writing – review and editing, project administration, funding acquisition, supervision

## Conflicts of interest

The authors declare no conflicts of interest.

## Data Availability

No primary research results, software, code or data were generated or analysed as part of this perspective.
